# Supplementary material for: Identification of photoperiod-induced specific miRNAs in the adrenal glands of Sunite sheep (Ovis aries)
Source: Front Vet Sci. 2022 Jul 22;9:888207. doi: 10.3389/fvets.2022.888207 (PMC9354845; doi:10.3389/fvets.2022.888207)
Supplement: Supplementary file 1 [file Data_Sheet_1.ZIP › Supplementary file/miRDeep2 file.zip!/miRDeep2 file/Know_Structure/SPLP42A3/oar-mir-26a.pdf]

miRBase precursor : oar-mir-26a
Total read count : 1118206
oar-miR-26a read count : 1118128
remaining reads : 78

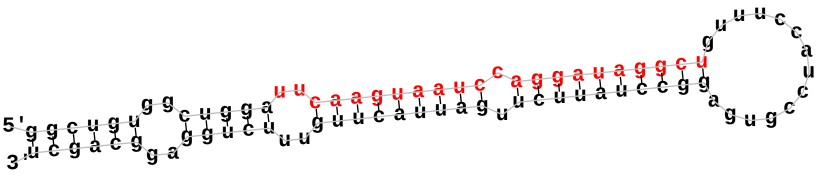

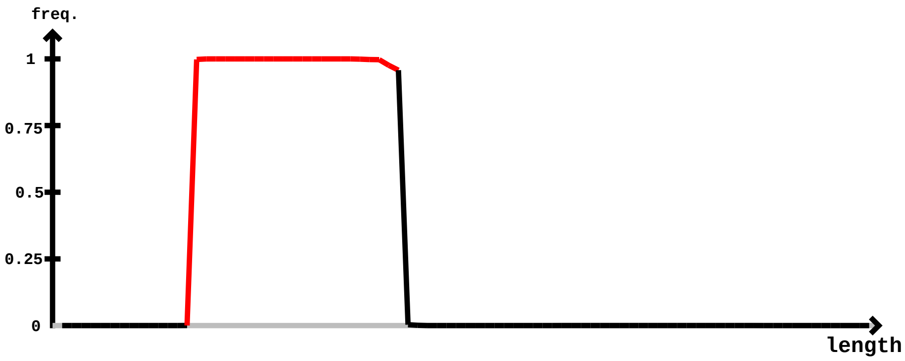


```
                  oar-miR-26a
5'- ggcuguggcuggauucaaguaauccaggauaggcuguuuccauccgugaggccuauucuugauuacuuguuucuggaggcagcu   -3'   exp
    ((((((..(((((..(((((((((.((((((((((..............)))))))))).)))))))))..)))))..))))))   reads   mm   sample
    ...........auucaaguaauccaggauagg....................................................   4   0   seq
    ...........auucaaguaauccaggauaggc...................................................   7   0   seq
    ...........auucaaguaauccaggauaggcu..................................................   234   0   seq
    ...........Guucaaguaauccaggauaggcu..................................................   41   1   seq
    ............uucaaguaauccaggCu.......................................................   1   1   seq
    ............uucaaguaauccaggaG.......................................................   10   1   seq
    ............uucaGguaauccaggau.......................................................   6   1   seq
    ............uucaaguGauccaggau.......................................................   1   1   seq
    ............uucaaguaCuccaggau.......................................................   1   1   seq
    ............uAcaaguaauccaggau.......................................................   9   1   seq
    ............uucaaAuaauccaggau.......................................................   1   1   seq
    ............uucaUguaauccaggau.......................................................   3   1   seq
    ............uucaaguaauccaggaA.......................................................   22   1   seq
    ............uucaaguaauccaggau.......................................................   1010   0   seq
    ............uucaaAuaauccaggaua......................................................   2   1   seq
    ............uucaGguaauccaggaua......................................................   5   1   seq
    ............uucaaguaauccaggaua......................................................   1638   0   seq
    ............uucaaguaauccaggauaC.....................................................   2   1   seq
    ............uucaaguaauccaggauag.....................................................   581   0   seq
    ............uucaaguaauccaggauagg....................................................   23670   0   seq
    ............uucaaguaauccaggauaggc...................................................   20306   0   seq
    ............uucaaguaauccaggauaggcu..................................................   1064678   0   seq
    ............uucaaguaauccaggauaggcug.................................................   2605   0   seq
    ............uucaaguaauccaggauaggcugu................................................   1057   0   seq
    ............uucaaguaauccaggauaggcuguG...............................................   1   1   seq
    ............uucaaguaauccaggauaggcuguu...............................................   5   0   seq
    ............uucaaguaauccaggauaggcuguuucc............................................   1   0   seq
    .............ucaaguaauccaggaua......................................................   1   0   seq
    .............ucaaguaauccaggauagg....................................................   31   0   seq
    .............ucaaguaauccaggauaggc...................................................   29   0   seq
    .............ucaaguaauccaggauaggcu..................................................   2021   0   seq
    .............ucaaguaauccaggauaggcug.................................................   9   0   seq
    .............ucaaguaauccaggauaggcugu................................................   10   0   seq
    ..............caaguaauccaggauagg....................................................   7   0   seq
    ..............caaguaauccaggauaggc...................................................   3   0   seq
```

oar-miR-26a

```
ggcuguggcuggauucaaguaauccaggauaggcuguuuccauccgugaggccuauucuugauuacuuguuucuggaggcagcu
...............caaguaauccaggauaggcu.................................................    89    0    seq
................Caguaauccaggauagg...................................................    2     1    seq
................aaguaauccaggauagg...................................................    2     0    seq
................aaguaauccaggauaggc..................................................    2     0    seq
................aaguaauccaggauaggcu.................................................    22    0    seq
................................................aggccuauucuugauuacuuguu.............    2     0    seq
...................................................ccuauucuugauuacuuguu.............    8     0    seq
...................................................ccuauucuugauuacuuguuu............    34    0    seq
...................................................ccuauucuugauuacuuguuuc...........    25    0    seq
...................................................ccuauucuugauuacuuguuucu..........    4     0    seq
....................................................cuauucuugauuacuuguuu............    2     0    seq
....................................................cuauucuugauuacuuguuuc...........    1     0    seq
....................................................cuauucuugauuacuuguuucu..........    1     0    seq
```
